# Evaluation of Unsupervised Deformable Image Registration using CNN and ViT on 4D-CT

**DOI:** 10.2174/0115734056385097251010051841

**Published:** 2025-10-22

**Authors:** Peizhi Chen, Jialan Wang, Yifan Guo, Yan Wang

**Affiliations:** 1 College of Computer and Information Engineering, Xiamen University of Technology, Xiamen, China

**Keywords:** Respiratory motion modeling, Evaluation, Deformable image registration, Convolutional neural networks, Vision transformer, Medical image analysis

## Abstract

**Introduction:**

Deformable image registration is essential in medical image analysis. The state-of-the-art approaches are unsupervised methods based on convolutional neural networks (CNN) and vision transformers (ViT). While CNNs perform well in extracting local features, ViTs perform better in extracting global features.

**Objective:**

This study aimed to compare the performance of CNN and ViT in unsupervised deformable image registration.

**Method:**

We have proposed a unified registration framework and evaluated both architectures. Experiments have been conducted using 4D-CT.

**Results:**

The results have shown ViT-based registration to achieve superior performance compared to CNN-based methods.

**Conclusion:**

The findings have indicated vision transformer architectures to be more effective than convolutional networks for unsupervised deformable registration on 4D-CT data.

## 1 INTRODUCTION

Deformable image registration aims to establish a non-linear mapping between two images. Image registration on 4D-CT is a critical step for respiratory motion modeling, which plays a vital role in analyzing lung motion in various applications, such as guided radiotherapy [1], surgical navigation [2], and motion compensation [3]. Complexdefor-mation in 4D-CT, particularly due to the significant changesduring the inhalation and exhalation phases of breathing, makes registration a challenging problem.

Existing image registration methods can be categorized into two classes: optimization-basedanddeep learning-based.Optimization-based image registration methods formulate image registration as an optimization problem, such as Elastix [4], HAMMER [5], SyN [6], and image matching [7-10]. Nonetheless, these methods can be time-consuming and are not appropriate for time-sensitive applications, such as guided radiotherapy [1].

Deep learning-based methods compute the mapping regressively using neural networks. They can be classified into two categories: supervised-based and unsupervised-based, depending on whether they require supervision information. Supervised-based methods depend on ground-truth deformation fields provided by optimization-based methods [11] or simulated deformations [12], which can limit their performance. In contrast, unsupervised-based methods do not require ground-truth deformation.

Most unsupervised-based methods are constructed on U-Net [13] or its variants [14, 15], which is an encoder-decoder structure with skip connections. Convolutional neural network (CNN) is among the most widely used neural networks. For example, Balakrishnan *et al*. [16, 17] proposed VoxelMorph, an unsupervised image registration method based on U-Net networks that do not require a supervised signal. Similarly, Chen *et al*. [18-20] proposed Dlung using a U-Net structure with CNN.

Alternatively, vision transformer (ViT) [21] is a neural network based on an attention mechanism that can consider global information. Chen *et al*. [22] proposed a ViT-based image registration framework. Chen *et al*. [23] proposed an improved ViT-based image registration framework.f

The performance of image registration using CNN and ViT on 4D-CT is still an area of ongoing research. CNN can capture local features, while ViT has long-range dependencies and can represent global deformations well. Yet, the evaluation of these two approaches in 4D-CT image registration has not yet been performed.

In this paper, we have evaluated unsupervised deformable image registration using CNN and ViT on 4D-CT. We have illustrated the framework of unsupervised deformable image registration. Both CNN and ViT can be applied in the framework. Then, we have defined both the detailed and rough evaluation metrics. We have conducted experiments using 4D-Lung and DIR-Lab. We have also proposed an improved method, named Dlung+. Results have demonstrated ViT to be better than CNN in unsupervised deformable image registra-tion. The contributions of this paper are listed as follows:

(1) An experimental comparison of unsupervised deformable image registration on 4D-CT using CNN and ViT.

(2) An improved unsupervised deformable image registration based on ViT, named Dlung+, and a demonstration of its effectiveness.

## 2 METHODS

A three-step framework has been constructed to build an unsupervised deformable image registration platform, as shown in Fig. (**1**). First, neural networks have been introduced to model the deformation field between the moving and fixed images. Then the moving image has been deformed to the moved image using a spatial transform. Finally, the loss function has guided the training, as shown by the dashed line. To evaluate the performance, the evaluation metrics have also been illustrated.

### 2.1 Neural Networks

Neural networks are utilized to model the deformation field. The nature of the field requires the model to represent a dense function. In other words, neural networks are a good choice due to their ability to model complex functions to any precision.

CNN and ViT are two prevalent neural networks.

CNN is effective at capturing local features, while it has limitations in global feature extraction. Conversely, ViT has long-range attention, while its ability for local feature extraction is limited.

To evaluate the performance of CNN and ViT in deformable image registration, we have employed a CNN-based method, namely VoxelMorph, and a ViT-based method, namely Vit-V-Net.

### 2.2 Spatial Transform

Optimizing the dense displacement field *via* the gradient-descent method can be challenging. To facilitate regular gradient-descent optimization, we have employed the widely used spatial transform layer to parameterize the displacement field so that it can be calculated using gradient descent optimization [24].

### 2.3 Loss Function

We have applied two conventional loss functions to drive the training: similarity and smoothness terms.

The similarity term is as follows:

**Table:** 

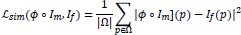	(1)

Where, Ω is the image domain.

The smoothness term is as follows:

**Table:** 

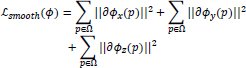	(2)

where, *p* is the voxel location and *φ* is the deformation domain.

### 2.4 Evaluation Metrics

We have evaluated the Dice score on two segmentations from the moved and fixed images to provide a detailed evaluation [25]. The Dice score between segmentation *I_A_* and *I_B_* has been calculated, and the higher the Dice score, the better.

Furthermore, we have evaluated the mean-squared difference (MSD), and the lower the MSD, the better.

**Table:** 

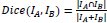	(3)

In addition to the Dice score and MSD, we have evaluated the number of folding pixels for a rough evaluation. We have also calculated the Jacobian determinant of every pixel, where *p* denotes each voxel of the deformation field *φp*. We have as well calculated the number of the negative *Jacdet* in every pixel. We have abbreviated Jacdet as |*J*| in the paper. |*J*| < 0 represents the number of folding pixels. The lower the number of |*J*| < 0, the better.

**Table:** 

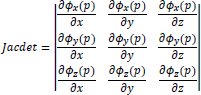	(4)

## 3 EXPERIMENTAL EVALUATION

### 3.1 MATERIAL

We conducted experiments on two datasets, 4D-Lung [26-29] and DIR-Lab [30, 31]. The 4D-Lung dataset consists of images acquired from 20 patients with the same device and settings. We used images from 15 patients for training and 5 for testing. In the DIR-Lab dataset, there were 300 manually identified feature locations on the extreme phase images. We did not train the model on DIR-Lab. Instead, we used the trained model from 4D-Lung to register CT images in the DIR-Lab dataset.

For both datasets, we employed a three-step preprocessing approach. First, we segmented lungs using a threshold; second, we resampled the segmented lungs to 160*192*224, and third, we normalized voxels whose HU values ranged from -1000 to 400 to [0,^1^].

### 3.2 IMPLEMENTATION

We performed an accuracy evaluation using the state-of-the-art methods, including optimization-based methods, such as SyN [6] and Elastix [4], and deep learning-based models, including VoxelMorph [32] and ViT-V-Net [22]. We used the public code for baseline methods.

Furthermore, building upon the pioneering work of Dlung [18], we have presented an improved method incorporating ViT and diffeomorphic registration, named Dlung+. Dlung + retains the same framework as Dlung, while the difference lies in replacing CNN with ViT to enhance the global feature extraction ability. We implemented Dlung+ using PyTorch. We trained Dlung+ using an NVIDIA RTX 3090 GPU. The training stage involved 1500 iterations using the Adam optimizer. The learning rate was initially set to 10^-4^.

In addition, for both CNN and ViT, we evaluated the performance of the squaring-scaling layer for diffeomorphic registration.

## 4 RESULTS

### 4.1 Results on 4D-Lung

We have made a comparison on the 4D-Lung dataset, as shown in Table **1**.

In Table **1**, it can be seen that ViT-based methods (ViT-V-Net and Dlung+) achieved a more accurate result than the CNN-based methods (VoxelMorph and Dlung) on the Dice scores. Dlung+ achieved the highest Dice score, and had fewer negative numbers of |*J*| than ViT-V-Net. In terms of running time and computational efficiency, the deep learning-based methods were significantly faster than the optimization-based methods.

We have further presented the results in the form of a boxplot, as depicted in Fig. (**2**).

In Fig. (**2**), the Dice score comparison is provided. Deep learning-based methods can be observed to be more accurate than optimization-based methods. It is evident that ViT-based methods outperformed CNN-based methods, while Dlung+ achieved the highest Dice score.

### 4.2 Results on DIR-Lab

We also evaluated the mean-squared difference (MSD) in the DIR-Lab datasets [30, 31] with manually labeled landmarks. The result is presented in Table **2**.

In Table **2**, ViT-based methods can be observed to provide a lower MSD than the CNN-based methods. Notably, Dlung+ achieved a slightly better MSD than ViT-V-Net.

We have further presented the results in the form of a box plot, as depicted in Fig. (**3**).

## 5 DISCUSSION

In this study, we have evaluated the accuracy performance of CNN and ViT and proposed an improved unsupervised deformable image registration platform on 4D-CT, named Dlung+. We have evaluated two optimization-based methods (SyN [6] and Elastix [4]) and four deep learning-based methods (VoxelMorph [32], Dlung [18], ViT-V-Net [22], and Dlung+) using the Dice score [25], Jacobian determinant, and mean-squared displacement (MSD) measurement. Deep learning-based methods have been found to outperform optimization-based methods. It may be due to the strong fitting ability of neural networks. It is worth noting that among the deep learning-based methods, ViT-based methods (ViT-V-Net and Dlung+) have been found to be better than the CNN-based methods (VoxelMorph and Dlung). It may be mainly because the long-range dependencies can capture global features. Our preliminary hypothesis suggests that image registration for 4D-CT demands greater emphasis on extracting global features. The accuracy of Dlung+ has been found to be comparable to state-of-the-art deep learning methods.

## CONCLUSION

In this study, we have evaluated the performance of unsupervised deformable image registration based on CNN and ViT on 4D-CT, and presented an improved method based on ViT, named Dlung+. Our experiments have demonstrated ViT to achieve a more accurate performance than CNN across both detailed and rough evaluation metrics in unsupervised deformable image registration on 4D-CT. ViT has been demonstrated to be more effective for 4D-CT registration than CNN. This finding is consistent with the success of advanced attention mechanisms in other complex spatio-temporal vision tasks, such as video-based re-identification [33] and multi-modal reasoning [34]. It aligns with the ongoing trend of applying innovative engineering solutions to enhance precision in clinical procedures [35].

## Figures and Tables

**Fig. (1):**
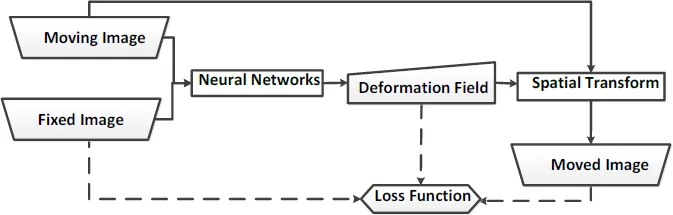
Framework of unsupervised deformable image registration.

**Fig. (2):**
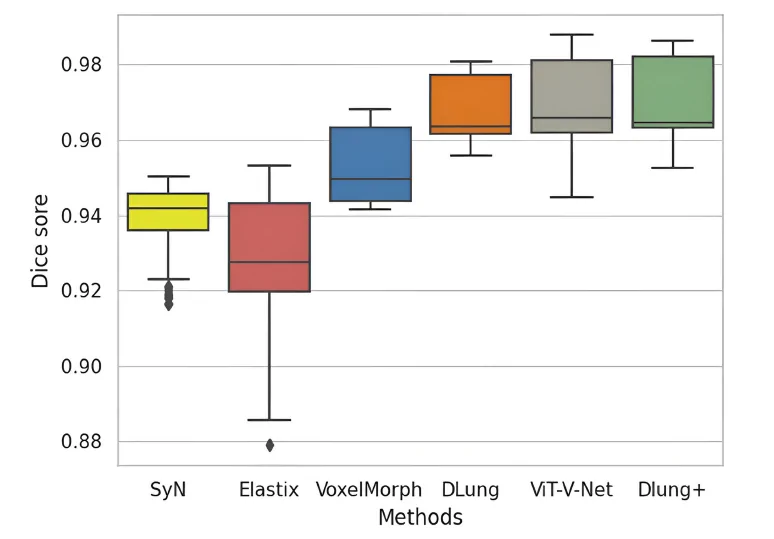
Dice score comparison on 4D-Lung dataset.

**Fig. (3):**
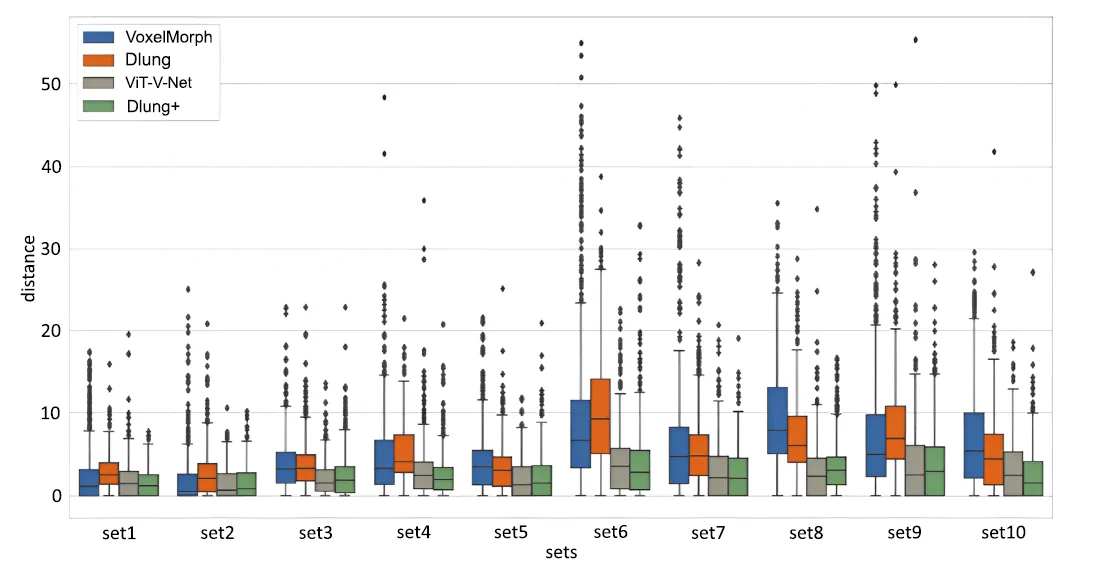
Comparison made on the DIR-Lab dataset using mean-squared difference (MSD).

**Table 1:** Accuracy comparison on the 4D-Lung dataset. We computed the Dice score on the lung parenchyma. The number of negative Jacobian determinants reflected the number of folding pixels.

Methods	Dice score	**|** *J* **|** **<** 0	Runtime (seconds)
SyN^1^	0.938(0.010)	0	25.05
Elastix^2^	0.927(0.019)	193.6(568.5)	34.38
VoxelMorph^3^	0.953(0.010)	154.8(255.4)	0.37
Dlung [18]	0.968(0.009)	109.08(164.8)	0.37
ViT-V-Net^4^	0.968(0.014)	48484.5(69500.8)	0.21
Dlung+	0.970(0.011)	143.3(201.2)	0.23

**Table 2:** Comparisons made on the DIR-Lab dataset using mean-squared difference (MSD). We computed the registration bidirectionally. The result is shown in mean (variance).

-	S1	S2	S3	S4	S5	S6	S7	S8	S9	S10
Original	3.89 (2.78)	4.34 (3.9)	6.94 (4.05)	9.83 (4.86)	7.48 (5.51)	10.89 (6.9)	11.03 (7.4)	15.00 (9.00)	7.92 (3.97)	7.30 (6.34)
VoxelMorph	2.35 (2.80)	1.91 (3.05)	3.97 (3.34)	4.49 (4.41)	4.14 (3.66)	9.30 (8.20)	6.27 (6.51)	9.33 (6.15)	6.78 (6.07)	6.75 (6.17)
Dlung	2.76 (2.39)	2.67 (2.90)	3.85 (2.91)	5.25 (3.44)	3.45 (2.97)	10.32 (6.15)	5.34 (4.09)	7.40 (4.80)	8.03 (5.66)	5.15 (4.47)
ViT-V-Net	1.88 (2.03)	**1.38** **(1.76)**	**2.09** **(2.05)**	3.12 (3.67)	**1.98** **(2.18)**	**4.07** **(3.84)**	2.93 (3.28)	**2.99** **(3.21)**	3.96 (4.82)	3.28 (3.63)
**Dlung+**	**1.46** **(1.4)**	1.48 (1.82**)**	2.42 (2.72**)**	**2.44** **(2.53)**	2.35 (2.82)	4.03 (4.77)	**2.85** **(2.93)**	3.37 (3.06)	**3.83** **(4.15)**	**2.59** **(3.25)**

## Data Availability

All the data and supporting information are provided within the article.

## Footnotes

1 The code can be obtained from https://github.com/ANTsX/ANTsPy

2 The code can be obtained from https://github.com/SuperElastix/SimpleElastix

3 The code can be obtained from https://github.com/junyuchen245 /ViT-V-Net_for_3D_Image_Registration_Pytorch

4 The code can be obtained from https://github.com/voxelmorph/voxelmorph

## LIST OF ABBREVIATIONS

CNN: Convolutional neural network
ViT: Vision transformers
